# DNA-Related Pathways Defective in Human Premature Aging

**DOI:** 10.1100/tsw.2002.226

**Published:** 2002-05-07

**Authors:** Vilhelm A. Bohr

**Affiliations:** Laboratory of Molecular Gerontology, National Institute on Aging, National Institutes of Health, 5600 Nathan Shock Dr., Baltimore, MD 21224, USA

**Keywords:** aging, premature aging, Werner syndrome, Cockayne syndrome, DNA repair

## Abstract

One of the major issues in studies on aging is the choice of biological model system. The human premature aging disorders represent excellent model systems for the study of the normal aging process, which occurs at a much earlier stage in life in these individuals than in normals. The patients with premature aging also get the age associated diseases at an early stage in life, and thus age associated disease can be studied as well. It is thus of great interest to understand the molecular pathology of these disorders.

## HUMAN PREMATURE AGING DISORDERS

A number of premature aging disorders have been described in humans. In patients with these disorders, aging-like symptoms and age-associated diseases appear much earlier than in the average normal individual; thus, the premature aging disorders are useful models for the study of the aging process. *Werner syndrome* (WS) is the most characterized premature aging disorder. Patients with WS have a large number of signs and symptoms of normal aging at a younger age than normal individuals. However, not all symptoms of WS resemble the normal aging process, and WS is best described as a segmental progeroid disorder. The premature aging disorders include the DNA-repair-defective disease *xeroderma pigmentosum* (XP)*,* which includes seven complementation groups (separate genetic disorders). In this condition, the deficiency of the DNA repair pathway, nucleotide excision, leads to a severely increased incidence of cancer. In *Cockayne syndrome,* there is also a DNA repair defect, and in addition to the features of premature aging, these individuals have severe neurological deficits. *Rothmund-Thompson* syndrome, *Hutchinson-Guilford,* and *progeria* are other examples of this category of disorders.

All of these diseases are very rare conditions in the general population. In several cases, the disorders are associated with a mutation in a single gene, which has by now been identified, cloned, and characterized. This means that molecular biochemical experimentation can be done. Complementation assays with transfected mutant cell lines or purified proteins added to extracts from mutant cell lines can be used to study the basis of molecular genetic defects.

## WERNER SYNDROME (WS) AND WERNER PROTEIN (WRNp)

Werner syndrome (WS) is a homozygous recessive disease characterized by early onset of many aspects of normal aging, such as wrinkling of the skin, graying of the hair, cataracts, diabetes, and osteoporosis. Cancers, particularly sarcomas, have been seen in these patients with increased frequency. The symptoms of WS begin to appear around the age of puberty, and most patients die before age 50.

WS is caused by mutations in a gene (*WRN*) belonging to the RecQ family of DNA helicases[1]. The WRN protein (WRNp) has been demonstrated biochemically to be a DNA-unwinding enzyme by several laboratories[2,3], including ours (see below). The enzyme is a DNA-dependent ATPase and catalyzes strand displacement in a nucleotide-dependent reaction. WRNp has more than one enzymatic activity. It has recently been shown that it also has a 3'-5' exonuclease activity[4,5,6]. Thus, WRNp has helicase, exonuclease, and ATPase activities.

The precise molecular deficiencies involved in the clinical phenotypes of WS are only partially understood. The phenotypical changes are shown in Table 1. The well-documented genomic instability of WS may point to a defect in replication or recombination[7]. WS cells exhibit premature replicative senescence and delayed S-phase progression in some cell lines[8]. A defect in recombination is suggested by the hyper-recombinant phenotype observed in some WS cell lines. WS cell lines are also hypersensitive to drugs that cause DNA interstrand cross-links[9,10]. These cross-links are repaired by recombinational pathways. Moreover, the *WRN* homologs in yeast, *Sgs*1 (*Saccharomyces cerevisiae*) and *rqh1* (*Schizosaccharomyces pombe*), as well as the bacterial homolog *recQ*, all suppress illegitimate recombination[11]. WRNp, *in vitro,* also has antirecombinase activity[12]. Thus, there is evidence that WRNp functions as an antirecombinase.

Some evidence suggests a role for the WRNp in DNA repair. This notion is supported by a report that WS cells are sensitive to the carcinogen 4-nitroquinoline 1-oxide (4-NQO)[13]. However, WS cells do not exhibit hypersensitivity to UV light or other DNA-damaging agents, suggesting that a repair deficiency in WS is subtle and perhaps not the primary defect in the disease. A fine-structure DNA repair defect in lymphoblast but not fibroblast WS cell lines was reported by this laboratory using the gene-specific and strand-specific repair assays[14].

In addition to repair or replication defects, evidence from this laboratory suggests that WS cells are defective in transcription[15]. This laboratory hase used a variety of WS lymphoblast cell lines that carry homozygous mutations in the *WRN* gene to assess the role of *WRN* in transcription. Transcription was measured both *in vivo*, by [$^3$H]-UTP incorporation in the chromatin of permeabilized cells, and *in vitro*, by using a plasmid template with an RNA polymerase II–specific promoter. The transcription efficiency in different WS cell lines was found to be reduced to 40–60% of that observed in normal cells using both assays[15]. This defect can be complemented by the addition of normal cell extract to the chromatin of WS cells. Furthermore, the addition of purified WRNp to the *in vitro* transcription assay stimulates RNA polymerase II–driven transcription. These observations suggest that the WRNp may act as a general activator of RNA polymerase II transcription.

**TABLE 1**
**Cellular Defects in WS**

| | |
|---|---|
| *Genomic instability* | *Replication* |
| - Chromosomal rearrangements | - Reduced replicative lifespan |
| - Large spontaneous deletions | - Extended S-phase |
| *Telomere maintenance* | - Reduced frequency of initiation sites |
| - Shortened telomeres | *DNA repair* |
| - SGS1 localization to telomeres | - Hypersensitivity to 4-NQO |
| *Recombination* | - Hypersensitivity to DNA cross-linking agents |
| - Hyper-recombination | - Reduced repair of psoralen cross-links |
| *Transcription* | - Hypersensitivity to camptothecin |
| - RNA polymerase II transcription | - Reduced telomeric repair |
| *Apoptosis* | - Reduced transcription-coupled repair |
| - Attenuated p53-mediated apoptosis | |

Our laboratory and others[2,3] have demonstrated that WRN helicase can proficiently unwind short DNA duplexes (~30 bp) in a reaction dependent on nucleoside triphosphate hydrolysis. Unwinding of long DNA duplexes ($\geq$250 bp) by WRN helicase is dependent on the presence of human Replication Protein A (RPA)[16].

## Werner Protein Interactions and Pathways

In recent years there has been a great deal of interest in the functions of WRNp. Biochemical properties and interactions of WRNp have been studied. The clarification of these interactions is important in our quest to understand in which pathways and processes WRNp participates. A list of reported protein interactions is shown in Table 2. One of the first established physical and functional interactions of WRNp was with RPA[16]. As mentioned, RPA is required for WRNp to unwind large DNA duplexes. Since RPA participates in DNA replication and DNA repair, this suggests that WRNp may also participate in both of those processes. It was later observed that the tumor suppressor protein p53 also binds to WRNp[17], and p53 is, incidentally, known to interact with RPA. Evidence from this laboratory shows that the physical interaction between WRNp and p53 is also associated with a functional interaction. p53 inhibits the exonuclease activity of WRNp directly[18] and thus may have a function in its regulation. This may imply that these proteins participate in a common pathway. p53 is known to participate in various signal transduction pathways, particularly in cell cycle regulation and apoptosis. Interestingly, WS cells have an attenuated apoptosis process[17], supporting the notion that p53 and WRNp both participate in the apoptotic process.

There is evidence for a role of WRNp in DNA replication. WS cells have been noted to have a prolonged S-phase[8], and the WRNp has been identified as a component of the replication complex in mammalian cells[19]. In addition, recent studies have shown that WRNp binds to and stimulates one of the major DNA polymerases, DNA polymerase $\delta$[20,21]. WRNp also interacts physically with another replication protein, proliferating cell nuclear antigen (PCNA), and the functional consequence of this interaction is not yet understood. The previously discussed RPA interaction also supports the notion that WRNp functions in replication. Taken together with the observation that WS cells have prolonged S-phase, it is likely that WRNp plays an important role in replication. This could be by resolving complex DNA structures during replication or by affecting the mobility of replication forks after exposure of cells to DNA damage.

**TABLE 2**
**WRN Protein Interactions**

*Physical Interactions*

| | |
|---|---|
| RPA | (Brosh *et al.*, 1999) |
| Ku 80/70 | (Cooper *et al.*, 2000;Li and Comai, 2000) |
| p53 | (Spillare *et al.*, 1999;Blander *et al.*, 1999) |
| human polymerase δ (p50) | (Szekely *et al.*, 2000) |
| PCNA | (Lebel *et al.*, 1999) |
| Topoisomerase I | (Lebel *et al.*, 1999) |
| WHIP | (Kawabe *et al.*, 2000) |
| Ubc9 | (Kawabe *et al.*, 2000) |
| FEN-1 | (Brosh et al., 2000) |

*Functional Interactions*

| | | |
|---|---|---|
| RPA | *Stimulates WRN helicase* | (Shen *et al.*, 1998;Brosh *et al.*, 1999) |
| Ku 80/70 | *Stimulates WRN exonuclease* | (Cooper *et al.*, 2000;Li and Comai, 2000) |
| p53 | *Inhibits WRN exonuclease* | (Brosh *et al.*, 2001) |
| Δ polymerase | *Stimulates DNA synthesis by yeast polδ* | (Kamath-Loeb *et al.*, 2000) |
| FEN-1 | *Stimulates FEN-1 nuclease* | (Brosh *et al.*, in press) |

As mentioned above, WS cells have increased genomic instability, and this has led to much speculation about whether a DNA repair pathway might be defective in WS. Recombination, in general, occurs via two different pathways, one that is homologous (requiring sequence homology) and one that is not, nonhomologous end rejoining (NHEJ). WRNp interacts both physically and functionally with the Ku heterodimer protein[22], which is involved in NHEJ and also is localized at telomeric ends. Ku binds very strongly to WRNp, suggesting that this interaction represents an important pathway in WRNp functions. It then strongly stimulates the WRNp exonuclease activity[22]. WS cells are sensitive to agents that form DNA interstrand cross-links[9,10], and these adducts in DNA are repaired by DNA recombination.

The biochemical steps of NHEJ specifically involve DNA helicase and exonuclease functions[23], and thus there is a need in this process for both of the catalytic functions of WRNp. So far, *in vitro* studies have not identified a biochemical role for WRNp in NHEJ, but there is a good deal of effort on resolution of this problem in various laboratories at this time.

The genome instability in WS has been suggested to arise from a failure to resolve alternate DNA structures. Sequences prone to form triple helices are abundant in the human genome. This laboratory has tested the ability of WRN helicase to unwind such structures, and we have found that the enzyme is able to unwind a 3' tailed triple-helix DNA substrate in a reaction dependent on nucleoside triphosphate hydrolysis[24]. It is possible that triplex structures are more persistent in WS cells, and this may be a basis for the variegated translocation mosaicism seen in WS cells.

During recombinational events such as DNA repair of lesions that include both DNA strands (e.g., cross-links), intermediate structures are formed in DNA. One such intermediate is the Holliday structure, where homologous DNA molecules exchange strands. The 3' end of the newly synthesized DNA strand invades the daughter duplex structure of the intact DNA molecule. These structures can be generated experimentally *in vitro*, and the mechanism of their resolution can be examined. In such an experiment, it appears that the purified WRN enzyme resolves this structure[12]. WRNp binds to a single-stranded site in the Holliday structure where the ruvA protein also binds. This unwinding is ATP-dependent, suggesting that it is due to the helicase activity of WRNp. Recent studies indicate that WRN helicase efficiently unwinds Holliday junctions[25], suggesting a direct role of WRNp in recombination.

## Werner Syndrome Functions and Normal Aging

The WRNp is a member of the RecQ family of helicases, to which the yeast Sgs1 and human Bloom proteins also belong. However, WRNp is unusual in that it is the only human member of this family of proteins that contains an exonuclease activity in addition to the helicase function. Until very recently, there has been no information about WRNp interactions or about in which protein complexes it participates. In the above, we have discussed various protein interactions involving WRNp; and, while the interactions are listed in Table 2, the mapping of some of these protein binders to WRNp is shown in Fig. 1. The implications of these interactions are that WRNp probably participates in all the pathways listed in Fig. 2 and discussed above. WRNp has attracted a lot of interest, and information is accumulating rapidly about its roles. Soon we should know much more about its interactions and complex formation.

Recent studies suggest that WRN may also participate in pathways at telomeric ends. WS cells display some defects in telomere metabolism, including increased rates of telomere shortening[26] and deficiencies in repair at telomeres[27]. Furthermore, expression of telomerase in WS cell lines prevents premature replicative senescence[28] and reduces hypersensitivity to 4-NQO[29]. There is also recent evidence for WRN localization at telomeric ends in some human fibroblast cell lines *in vivo*[30]. In addition, the WRN yeast homologue Sgs1 was shown to participate in a telomerase-independent mechanism of telomere lengthening in yeast[30]. Therefore, WRN may function at telomeres by resolving secondary structure in order to allow access to replication, repair, and/or telomere-lengthening machinery.

Werner syndrome is a segmental progeria. This means that the disease is associated with a large number of clinical features that are seen in normal aging at a later stage in life. Whereas WS cannot be equated with the normal aging process, it is of significant interest that a mutation in one gene can lead to so many signs of aging. These include the increased frequency of age-associated diseases such as cancers and type II diabetes, and they include a large spectrum of phenotypic changes associated with the normal aging process[31]. Thus, insight into the molecular pathways involving the WRNp is bound to shed important light on the normal aging process and on why aging is accompanied by such a high level of associated disease.

## COCKAYNE SYNDROME

Cockayne syndrome (CS) is a rare, inherited human genetic disease categorized as a segmental progeria. Affected individuals suffer from postnatal growth failure resulting in cachectic dwarfism, photosensitivity, skeletal abnormalities, mental retardation, progressive neurological degeneration, retinopathy, cataracts, and sensorineural hearing loss[32,33]. Two complementation groups, CS-A and CS-B, have been identified, and the corresponding genes have been cloned[34,35]. The cellular phenotype of CS includes increased sensitivity to a number of DNA-damaging agents, including UV radiation, ionizing radiation, and hydrogen peroxide[36,37].

Nucleotide excision repair (NER) is a complex process that removes and repairs many types of DNA lesions. NER has two subpathways: (1) the transcription-coupled repair (TCR) pathway and (2) the global genome repair (GGR) pathway. The TCR pathway repairs lesions in the

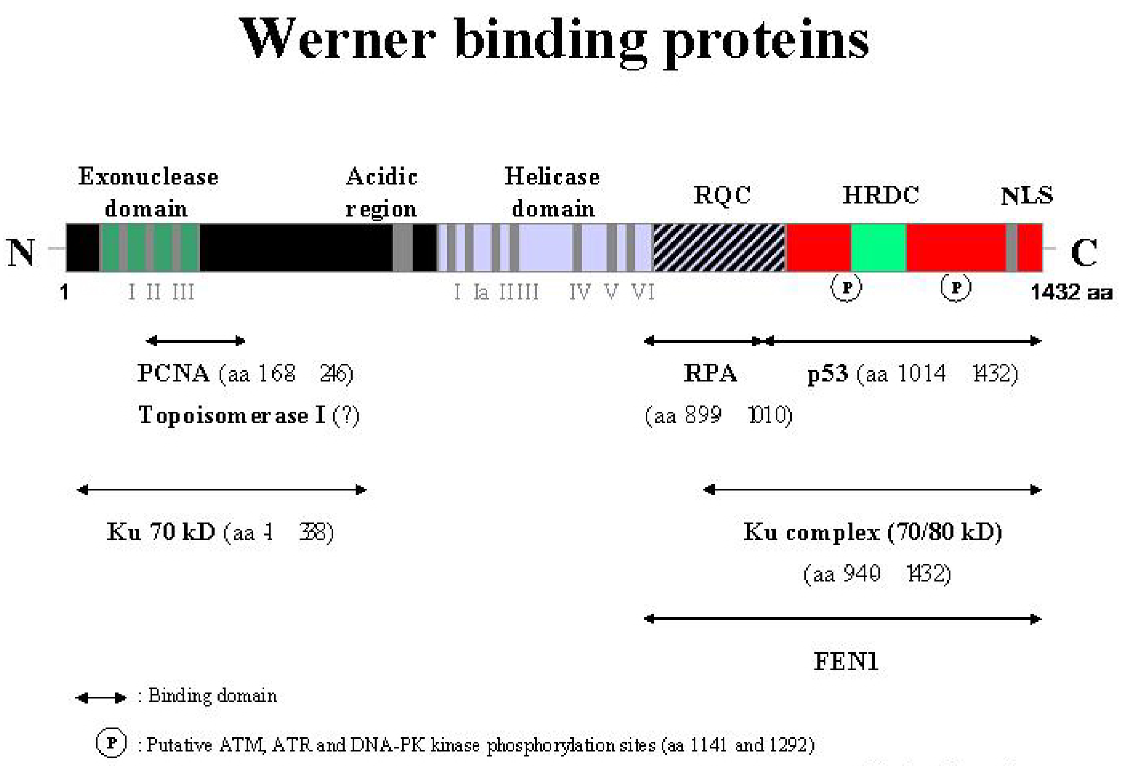

**FIGURE 1.** Werner protein interactors.

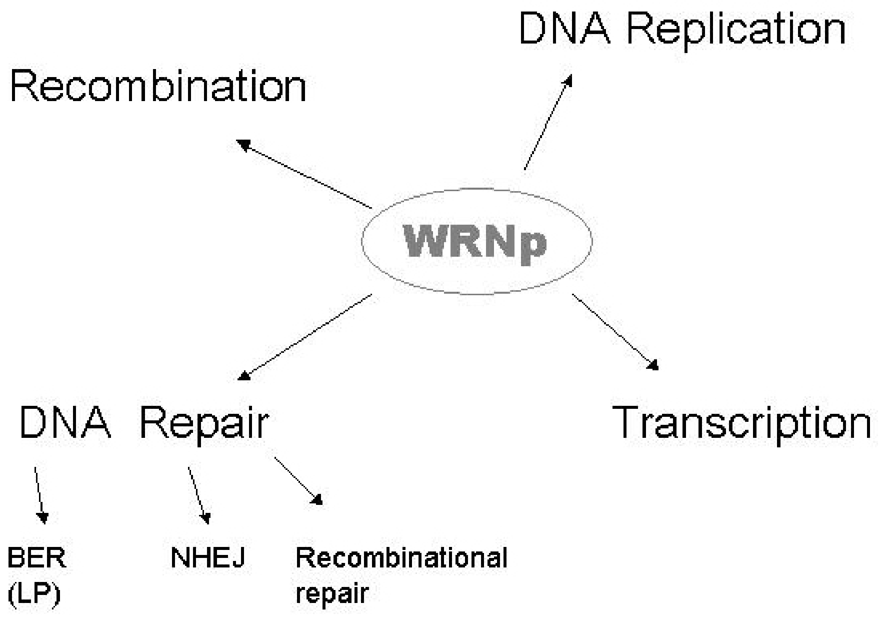

**FIGURE 2.** Processes in which Werner protein participates.

transcribed strand of transcriptionally active genes and is dependent on RNA polymerase II (RNA pol II)[38,39]. The GGR pathway removes lesions from genes that are transcriptionally active or inactive. CS cells are defective in TCR but proficient in GGR of UV-induced DNA damage[40]. A characteristic feature of CS cells is that they do not recover the ability to efficiently synthesize RNA after UV damage[41]; this phenotype is consistent with a defect in TCR.

The *CSA* and *CSB* genes have been cloned and their products characterized biochemically. The *CSA* gene product is a 44-kDa protein that belongs to the "WD repeat" family of proteins[35]. Members of this protein family are structural and regulatory proteins, but they do not have enzymatic activity. The *CSB* gene product is a 168-kDa protein[34] that belongs to the SWI/SNF family of proteins, which are DNA and RNA helicases with seven conserved sequence motifs[42]. In addition, CSB has an acidic amino acid stretch, a glycine-rich region, and two putative NLS sequences. CSB is a DNA-stimulated ATPase but is not able to unwind DNA in a conventional strand-displacement assay[43,44]. The human *CSB* cDNA complements the UV sensitivity and the delay in RNA synthesis recovery when transfected into the human CS-B–deficient cell line CS1AN.S3.G2[34] or the hamster CS-B–deficient cell line UV61[34]. Gene-specific TCR repair is also recovered when the human *CSB* gene is transfected into UV61 cells[45,46].

The precise molecular role of CSB is not clear at present. CSB may facilitate repair of active genes by recruiting DNA repair proteins to actively transcribed regions. *In vitro,* CSB exists in a quaternary complex with RNA pol II, DNA, and the RNA transcript, and ATP hydrolysis is required to form this complex[44]. This quaternary complex recruits another molecular complex that includes the transcription factor IIH (TFIIH) core subunits p62 and XPB[47]. TFIIH is a complex factor thought to promote local DNA unwinding during transcription initiation by RNA pol II and promoter escape, as well as in NER[48,49,50,51,52]. Coimmunoprecipitation studies demonstrate that XPG and CSB proteins interact[53]. XPG interacts with multiple components of TFIIH[53], and it is an endonuclease that plays a role in NER. *In vitro,* CSB interacts with CSA and with the NER damage recognition factor XPA[35,43]. These protein interactions support the hypothesis that CSB participates directly in TCR.

It is also possible that CSB indirectly stimulates TCR by facilitating transcription. Members of the SWI/SNF family are involved in transcription regulation, chromatin remodeling, and DNA repair, including such actions as disruption of protein–protein and protein–DNA interactions (reviewed in[54]). The *CSB* gene product could have a similar function. In fact, it is still a matter of debate whether CS is due to a primary defect in transcription or DNA repair[55,56]. The basal transcription level is lower in human CS-B lymphoblastoid cells and fibroblasts than in normal cells, even without exposure to DNA-damaging agents[57]. This transcription defect is complemented by normal cell extracts *in vitro* or by the wild-type *CSB* gene *in vivo*. In a reconstituted system, purified CSB protein enhances the rate of transcription by RNA pol II[58], suggesting that CSB may indirectly stimulate TCR by facilitating the process of transcription[58]. Thus, CSB may be a transcription elongation factor and a repair-coupling factor acting at the site of RNA pol II–blocking lesions, and the CS phenotype may arise from deficiencies in both transcription and DNA repair. The biological function of CSB in these different pathways may be mediated by distinct functional domains of the protein.

As described above, it is well-established that the *CSB* phenotype involves a defect in TCR of UV-induced DNA damage. However, evidence also suggests that TCR of oxidative damage may be affected by CSB[59]. Hydrogen peroxide and ionizing radiation induce a large variety of DNA damage, including oxidatively damaged bases and single- and double-strand DNA breaks.

**TABLE 3**
**DNA Repair Transcription, and Chromatin Structure in Cockayne Syndrome (CS)**

- **DNA Repair**
  - **TCR after UV (NER) defect (Venema et al., Evans et al.)**
  - **TCR after X-ray and oxidative damage (Cooper et al.)**
  - **Accumulation of base modifications (Stierum et al.)**
  - **Incision defect (BER) (Dianov et al.)**
  - **PCNA relocation defect (Balajee et al.)**
- **Transcription**
  - **Basal transcription defect *in vivo* (Balajee et al.)**
  - **Transcription defect *in vitro* (Dianov et al., Selby and Sancar)**
  - **CSB is an elongation factor (Sancar)**
  - **CSB binds to RNA polymerase II (Hoeijmakers)**
- **Chromatin Structure**
  - **Lack of nuclear matrix association (Mullenders)**
  - **Loose chromatin structure (Balajee et al.)**
  - **Chromatin remodeling (Citterio et al.)**
- **Apoptosis**
  - **Increased apoptosis**

It has been shown that primary CS-B cells are slightly more sensitive to $\gamma$-irradiation; and in addition, they are defective in strand-specific repair of ionizing radiation and hydrogen peroxide–induced thymine glycol[36,37]. Previously, this laboratory showed that the ability of CS-B cell extracts to carry out incision of 8-oxoguanine lesions *in vitro* is 50% of normal; in contrast, incision of uracil and thymine glycol were normal[60]. 8-Oxoguanine is a base modification caused by oxidative stress from environmental agents and from endogenous metabolic processes, and it is repaired mainly via base excision repair (BER)[61,62]. Further recent studies from this laboratory show that the CSB protein is involved in BER. We have established stably transfected human cell lines in which functional domains of the *CSB* gene have been mutated[63]. When certain helicase motifs are disrupted, there is a cellular defect in the incision of DNA containing 8-oxoG in cell extracts[63].

Understanding the exact role of CSB is quite challenging. Some of the cellular deficits, which also give clues to the pathways that involve CSB, are listed in Table 3. We are convinced that this important protein participates not only in transcription-coupled repair but also in BER, and that it also plays a role in transcription.
